# Optimization of Total Flavonoid Compound Extraction from *Gynura medica* Leaf Using Response Surface Methodology and Chemical Composition Analysis

**DOI:** 10.3390/ijms11114750

**Published:** 2010-11-22

**Authors:** Wei Liu, Yanying Yu, Ruzhen Yang, Chunpeng Wan, Binbin Xu, Shuwen Cao

**Affiliations:** 1 State Key Laboratory of Food Science and Technology, Nanchang University, Nanchang 330047, Jiangxi, China; E-Mails: nculiuwei@126.com (W.L.); lemonwan@126.com (C.W.); ncuxbb@126.com (B.X.); 2 Department of Chemistry, Nanchang University, Nanchang 330031, Jiangxi, China; E-Mail: yuyanying@ncu.edu.cn (Y.Y.); 3 College of Food Science and Engineering, Shandong Agricultural University, Tai’an 271018, Shandong, China; E-Mail: yangruzhen123@163.com

**Keywords:** *Gynura medica*, response surface methodology, flavonoid compounds, antioxidant activity

## Abstract

Optimization of total flavonoid compound (TFC) extraction from *Gynura medica* leaf was investigated using response surface methodology (RSM) in this paper. The conditions investigated were 30–60% (v/v) ethanol concentration (*X*_1_), 85–95 °C extraction temperature (*X*_2_) and 30–50 (v/w) liquid-to-solid ratio (*X*_3_). Statistical analysis of the experiments indicated that temperature and liquid-to-solid ratio significantly affected TFC extraction (*p* < 0.01). The Box-Behnken experiment design showed that polynomial regression models were in good agreement with the experimental results, with the coefficients of determination of 0.9325 for TFC yield. The optimal conditions for maximum TFC yield were 55% ethanol, 92 °C and 50 (v/w) liquid-to-solid ratio with a 30 min extraction time. Extracts from these conditions showed a moderate antioxidant value of 54.78 μmol quercetin/g dry material (DM), 137.3 μmol trolox/g DM for 1,1-diphenyl-2-picrylhydrazyl (DPPH) and 108.21 μmol quercetin/g DM, 242.31 μmol trolox/g DM for 2,2-azino-bis-(3-ethylbenzthiazoline-6-sulphonic acid) (ABTS^+^), respectively. HPLC-DAD-MS analysis showed that kaempferol-3-*O*-glucoside was the principal flavonoid compound in *Gynura medica* leaf.

## 1. Introduction

Flavonoid compounds, which are a large group of secondary metabolites in higher plants [1], are known to be responsible for antioxidant [2,3], anticancer [4,5], anti-inflammatory [6,7], hepatoprotective potential [8,9] and antibacterial activity [10,11]. Recently, epidemiological studies have strongly suggested that consumption of plant flavonoids contributes to reducing the risk or incidence of some cancers [12,13]. Also, more and more attention has been focused on the antioxidant activity of medicinal plants, fruits and vegetables that contain plentiful flavonoid compounds. However, the content of flavonoid compounds is influenced by many factors, including the genus, the place of the plant growth, the extraction conditions and technology [14].

*Gynura medica* (Compositae) is a newly found plant belonging to the genus *Gynura* [15]. The ethanol extract of *G. medica* had demonstrated hypoglycemic activity in animal models [16,17]. Many plants of the genus *Gynura* have been used for the treatment of bronchitis, pulmonary tuberculosis, pertussis, sore eyes, toothache, rheumatic arthralgia and diabetes in folk medicine [18]. They were also used as a nutriment vegetable by the local people in the southeast area of China. The chemical constituents of the genus *Gynura* include flavonoid, phenolic acid, cerebrosides, polysaccharide, alkaloids, terpenoids and sterols [18–21]. Phenolic acid and flavonoid are the major components of the *Gynura* genus. However, little information is available concerning the chemical constituents and pharmacologic activity of the plant. In particular, the flavonoid compounds in the *G. medica* leaf have not been fully elucidated.

Response surface methodology (RSM) is an effective statistic technique for optimizing complex processes. It has been successfully demonstrated that RSM can be used to optimize the TFC from many medicine plants [14]. In the present study, the influence of some extraction variables on TFC is conducted. A Box-Behnken experimental design was performed in order to optimize the optimal extraction conditions of TFC from *G. medica*. The free radical scavenging activity has been determined by DPPH and ABTS**^+^** methods. Meanwhile, the flavonoid compounds from *G. medica* were separated and identified by high-performance liquid chromatography-diode array detector-electrospray ionization-tandem mass spectrometry (HPLC-DAD-ESI-MS) analytical method, the quantification of those identified flavonoid compounds were conducted by HPLC using the kaempferol-3-*O*-glucoside as a standard reference.

## 2. Results and Discussion

### 2.1. Effect of Time on the Extraction Yield of TFC

**Figure 1.**
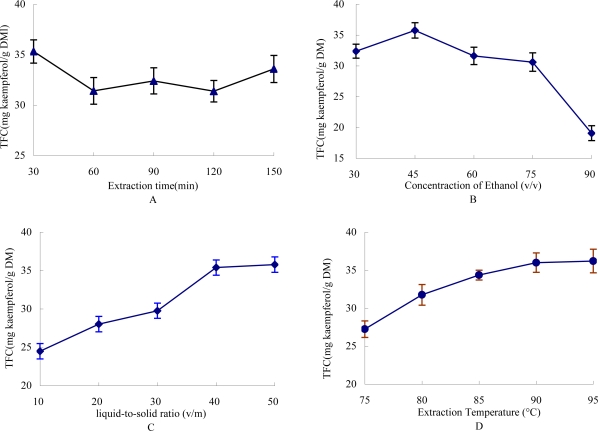
The effects of extraction parameters on TFC yield. (**A**) Effect of extraction time on TFC yield, the other extraction conditions were 45% ethanol, 40:1 liquid-to-solid ratio and 85 °C extraction temperature; (**B**) Effect of ethanol concentration on TFC yield, the other extract condition were 40:1 liquid-to-solid ratio, 85 °C extraction temperature and 30 min extraction time; (**C**) Effect of liquid-to-solid ratio on TFC yield, the other extract condition were 45% ethanol, 85 °C extraction temperature and 30 min extraction time; (**D**) Effect of extraction temperature on TFC yield, the other extract condition were 45% ethanol, 40:1 liquid-to-solid ratio and 30 min extraction time.

Extraction time is a factor that would significantly influence the extraction efficiency of TFC from medicine plants or edible vegetables. The effect of different times on extraction yield of TFC is shown in Figure 1A. Extraction was carried out at different times while other extraction parameters were kept constant (45% ethanol, 40:1 liquid-to-solid ratio and 85 °C extraction temperature). When extraction time varied from 30 min to 120 min, the yield of TFC was significantly decreased from 35.32 to 31.38 g kaempferol/g dry material (DM), while when time was increased to 150 min the yield of TFC was increased to 33.58 mg kaempferol/g DM. It may be that the increase in extraction time led to the degradation of flavonoid compounds, which indicated that 30 min was sufficient to obtain the TFC from *G. medica* leaves. Thus 30 min was picked as the favorable extraction time.

### 2.2. Effect of Ethanol Concentration on the Yield of TFC

The concentration of extraction solvent influences the efficiency of TFC: Generally lower concentration of ethanol is suitable for extraction of polar flavonoid compounds and higher concentration ethanol is suitable for extraction of non-polar flavonoid compounds. The effect of the concentration of ethanol on extraction yield of TFC is shown in Figure 1B. Extraction was carried out with different concentrations of ethanol (30–90%, v/v) while other extraction parameters were constant (40:1 liquid-to-solid ratio, 85 °C extraction temperature and 30 min extraction time). The TFC significantly increased from 32.37 to 35.76 mg kaempferol/g DM when the concentration of ethanol increased from 30% to 45%. However, as the concentration of ethanol continued to increase up to 90%, the TFC decreased to 19.07 kaempferol/g DM. The reason may be because the flavonoid glycosides were the major constituents of the plant; higher concentrationd of ethanol were adverse to extract the flavonoid glucosides.

### 2.3. Effect of Liquid-to-Solid Ratio on the Yield of TFC

The effect of liquid-to-solid ratio on the extraction yield of TFC is shown in Figure 1C. Extraction was carried out at different liquid-to-solid ratios (10–50, v/w) while other extraction parameters were constant (45% ethanol, 85 °C extraction temperature and 30 min extraction time). The extraction yields of TFC significantly increased from 24.48 to 35.39 mg kaempferol/g DM as the liquid-to-solid ratio increased within the range of 10–40 (v/w), due to the increase of the driving force for the mass transfer of the TFC. However, the extraction yields no longer significantly changed when the liquid-to-solid ratio continued to increase.

### 2.4. Effect of Extraction Temperature on the Yield of TFC

The effect of extraction temperature on the extraction yield of TFC is shown in Figure 1D. Extraction was carried out at different extraction temperatures (75–95 °C) while other extraction parameters were constant (45% ethanol, 40:1 liquid-to-solid ratio and 30 min extraction time). The TFC extraction yields significantly increased from 27.24 to 36.21 mg kaempferol/g DM as the extraction temperature increased from 75 to 95 °C, due to the increasing extraction temperature promoting solvent extraction by enhancing both diffusion coefficients and the solubility of flavonoid content.

### 2.5. Optimization of the Yield of TFC

The TFC extraction from *G. medica* leaves was further optimized through the RSM approach. A fixed extraction time (30 min) was chosen. The coded and actual levels of the three variables in Table 1 were selected to maximize the TFC. 15 experiments were designated, in which 1–12 were factorial experiments and 13–15 were zero-point tests performed in triplicate to estimate the errors.

Table 2 shows the treatments with coded levels and the experimental results of TFC in *G. medica* leaves. The TFC yield ranged from 31.66 to 36.22 mg kaempferol/g DM. The maximum yield of TFC was recorded under the experimental conditions of *X*_1_ = 45%, *X*_2_ = 95 °C and *X*_3_ = 50. By applying multiple regression analysis on the experimental data, the response variable (TFC) and the test variables are related by the following second-order polynomial equation:

(1) $$\begin{array}{l} Y=35.97+0.38X_{1}+0.65X_{2}+1.15X_{3}+0.47X_{1}X_{2}+0.096X_{1}X_{3}+0.015X_{2}X_{3}\\ -2.06X_{1}^{2}-0.80X_{2}^{2}-0.52X_{3}^{2} \end{array}$$

Table 3 shows the analysis of variance (ANOVA) for the regression equation. The linear term and quadratic term (except for *X*_3_^2^) were significant (*p* < 0.05), while the interaction was not significant, indicating that the relationship between response variable (TFC) and the test variables was not simply a linear one. The lack of fit was used to verify the adequacy of the model. ANOVA for the lack of fit was not significant (*p* > 0.05) for the model, indicating that the model could adequately fit the experiment data.

The adequate precision value is a measure of the “signal (response) to noise (deviation) ratio”. A ratio greater than 4 is desirable. In this study, the ratio was found to be 13.9, which indicates an adequate signal and therefore the model is significant for the extracting process. The value of *R*_Adj_^2^ (0.9325) for the equation is reasonably close to 1, indicated a high degree of correlation between the observed and predicted values, therefore the model is suitable. A very low value of coefficient of the variance (C.V.) (1.19) clearly indicated a very high degree of precision and reliability of the experimental values.

**Figure 2.**
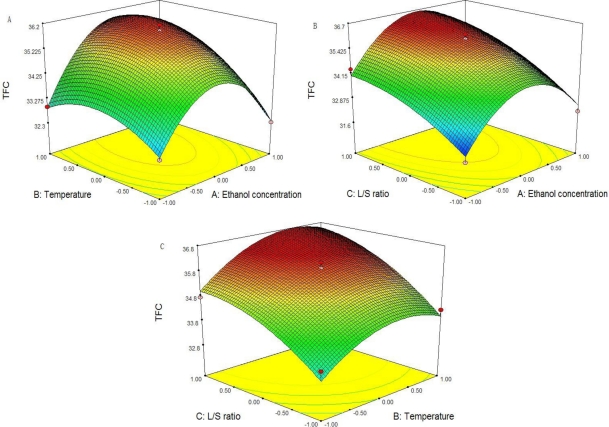
Three-dimensional plot of TFC. (**A**) Response plot of Ethanol concentration (*X*_1_) *vs.* Temperature (*X*_2_); (**B**) Response plot of Ethanol concentration (*X*_1_) *vs.* liquid -to-solid ratio (*X*_3_); Response plot of Temperature (*X*_2_) *vs.* liquid -to-solid ratio (*X*_3_).

Three-dimensional response surface plots are presented in Figure 2. An increase of temperature (*X*_2_) and liquid-to-solid ratio (*X*_3_) result in an increase of TFC to a maximum at a certain levels, while an increase of ethanol concentration (*X*_1_) results in an initial increase of TFC that then decrease when ethanol concentration continues to increase.

The optimal values of the selected variables were obtained by solving the regression equation. After calculation by Design-Expert software, the values thus obtained were *X*_1_ = 0.74, *X*_2_ = 0.71 and *X*_3_ = 1, with the corresponding *Y* = 36.215 mg kaempferol/g DM. Using RSM, the optimal conditions of TFC were an extraction of 55% ethanol, 92 °C extraction temperature and 50 (v/w) liquid-to-solid ratio with 30 min extraction time. To confirm these results, three triplicate tests were performed under optimized conditions. The TFC yield value was 36.19 ±0.09 (*n* = 3), which clearly showed that the model fitted the experimental data and therefore optimized the TFC extraction procedure from *G. medica* leaves.

### 2.6. DPPH and ABTS^+^ Cation Free Radical Scavenging Capacity

DPPH and ABTS^+^ are the two methods used extensively in estimation of the antioxidant capacity of pure compounds and plant extracts [22,23]. The free radical scavenging activity of *G. medica* leaf extracted with the optimum conditions were measured using these two methods. The samples were able to inhibit the activity of both DPPH and ABTS^+^ radicals in a dose-dependent manner (Tables 4 and 5). The calibration curves were obtained for trolox and quercetin. Results were expressed as μmol standard reference equivalents per g dry material. The *G. medica* leaf extract showed a moderate radical scavenging activity, which agreed with the moderate flavonoid compounds existing in the plant, the glucosides of kaempferol were the major flavonoid compounds existing in the plant (kaempferol-3-*O*-glucoside was the major compound of the plant). Meanwhile, kaempferol was a moderate antioxidant agent compared with quercetin. The structure of kaempferol comprises 4 hydroxy. The more hydroxy existing in the chemical structure, the higher the antioxidant activity exhibited.

### 2.7. HPLC-DAD-MS Analysis and Quantification of Flavonoid Compounds

**Figure 3.**
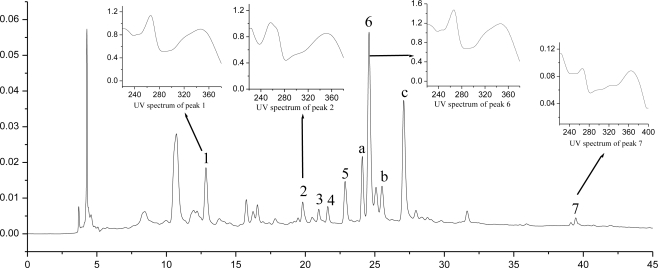
The HPLC-DAD chromatogram of *G. medica* leaf extract.

Seven peaks of flavonoid compounds were separated and identified by the MS value coupled with the UV spectrum data (Figure 3, Table 6). Peaks of 1, 4, 5 and 6 shared the same UV spectrum showing λ_max_ at 264 nm and 347 nm, suggesting that those were kaempferol glycosides [24,25]. Peak 1 yielded 609 [M − H]^−^, 611 [M + H]^+^, 449 [M + H − 162]^+^ and 287 [M + H – 162 − 162]^+^, kaempferol-3,7-di-*O*-*β*-d-glucoside was consistent with the above data and had been isolated from another *Gynura* plant previously [26]. Both peaks 4 and 5 yielded 593 [M − H]^−^, 595 [M + H]^+^, 449 [M + H − 146]^+^ and 287 [M + H − 146 − 162]^+^, kaempferol-3-*O*-robinobioside and kaempferol-3-*O*-rutinoside were consistent with the above data by examining the known kaempferol glycoside in the genus of *Gynura* [19,27]. The elution order in HPLC of kaempferol-3-*O*-robinobioside was prior to kaempferol-3-*O*-rutinoside as has been reported many times in the literature [28,29]. Thus, peak 4 and 5 were identified as kaempferol-3-*O*-robinobioside and kaempferol-3-*O*-rutinoside, respectively. Peak 6 yielded 447 [M − H]^−^, 449 [M + H]^+^ and 287 [M + H − 162]^+^, the dominant flavonoid compounds existing in *G. medica* was identified as kaempferol-3-*O*-*β*-d-glucoside. Peaks 2 and 3 shared the same UV spectrum, showing λ_max_ at 255 nm and 355 nm, suggesting those were quercetin glycosides [30]. Peak 2 yielded 609 [M − H]^−^, 611 [M + H]^+^, 465 [M + H − 146]^+^ and 303 [M + H – 146 − 162]^+^. Rutin was found present in many other *Gynura* plants [19,31], which was consistent with the above data. Peak 3 yielded 463 [M − H]^−^, 465 [M + H]^+^ and 303 [M + H − 162]^+^, quercetin-3-*O*-*β*-d-glucoside was consistent with the above data [32,33]. Peak 7 UV spectrum showed λ_max_ at 266 nm and 367 nm, yielding a quasimolecule ion at 285 [M − H]^−^ and 287 [M + H]^+^, which was identified as kaempferol. The quantification of the seven flavonoid compounds were carried out using external standard method and the results were expressed as mg kaempferol-3-*O*-*β*-d-gucoside/g dried material (Table 6). Kaempferol-3-*O*-*β*-d-gucoside was the major flavonoid compound of the plant, followed by kaempferol-3,7-di-*O*-*β*-d-glucoside and kaempferol-3-*O*-rutinoside. In a word, kaempferol glucoside derivatives were the major flavonoid constituents existing in the plant.

Meanwhile, many other components existed in the extracts (e.g., peaks a, b and c), which shared the same UV spectrum showing λ_max_ at 248 nm, 293 nm (sh) and 327 nm, suggesting that those were Dicaffeoylquinic acid derivatives (both of them gave a [M − H]^−^ ion at *m/z* 515, [M – H − 162]^−^ ion at *m/z* 353, and [M + H]^+^ ion at *m/z* 517, [M + H − 18]^+^ ion at *m/z* 499, [M + H – 162 − 192]^+^ ion at *m/z* 163, data not shown). This completely agrees with the literatures reporting that Dicaffeoylquinic acid derivatives commonly exist in *Compositae* plants [34].

## 3. Experimental

### 3.1. Sample Preparation and Chemicals

One kilogram of *Gynura medica* was obtained in March of 2010 from Huoshan districts, Anhui province, China. A voucher specimen (2010R01) was deposited at the Department of Chemistry, Nanchang University. *G. medica* leaves were dried at room temperature for three weeks and finely powdered in a knife mill. Trolox, quercetin, kaempferol and 1,1-diphenyl-2-picrylhydrazyl (DPPH) were purchased from Sigma Chemical Company (St. Louis MO, USA), 2,2-azino-bis-(3-ethylbenzthiazoline-6-sulfonic acid) (ABTS) was obtained from TCI-SU (Tokyo, Japan), potassium persulfate, aluminium choride, sodium acetate, and all solvents used were of analytical grade and purchased from Sinopharm Chemical Reagent Co., Ltd. (Shanghai, China). Visible spectra measurements were done using a UV-2450 spectrophotometer (Shimadzu, Japan).

### 3.2. Experimental Design

The extraction parameters were optimized using response surface methodology (RSM) [35]. A Box-Behnken experiment was employed in this regard. Ethanol concentration (*X*_1_), extraction temperature (*X*_2_) and liquid-to-solid ratio (*X*_3_) were chosen for independent variables. The range and center point values of three independent variables presented in Table 1 are based on the results of preliminary single factor experiments. The experimental design consists of 12 factorial experiments and three replicates of the central point (Table 2). TFC was selected as the responses for the combination of the independent variables given in Table 2. Three triplicate experiments were carried out at each experimental design point and the mean values were stated as observed responses. Experimental runs were randomized, to minimize the effects of unexpected variability in the observed responses. The variables were coded according to the following equation:

(2) $$x=(X_{i}-X_{0})/\Delta X$$

where *x* is the coded value, *X_i_* is the corresponding actual value, *X*_0_ is the actual value in the center of the domain, and Δ*X* is the increment of *X_i_* corresponding to a variation of 1 unit of *x*. The mathematical model corresponding to the Box-Behnken design is:

(3) $$Y=\beta_{0}+\sum_{i=1}^{3}\beta_{1}X_{i}+\sum_{i=1}^{3}\beta_{ii}X_{i}^{2}+\sum_{i=1}^{2}\sum_{j=1+1}^{3}\beta_{ij}X_{i}X_{j}+ɛ$$

where *Y* is the dependent variable (TFC), *β*_0_ is the model constant, *β_i_*, *β_ii_* and *β_ij_* are the model coefficients, and *ε* is the error. They represent the linear, quadratic and interaction effects of the variables. Analysis of the experimental design data and calculation of predicted responses were carried out using Design Expert software (Version 7.0.0, Stat-Ease, Inc., Minneapolis, MN). Additional confirmation experiments were subsequently conducted to verify the validity of the statistical experimental design.

### 3.4. Solvent Extraction

The single factors for solvent extraction procedures were set as follows: Firstly, the effect of extraction time on the extraction was investigated. *Gynura medica* leaf powder was put into a 100 mL round bottomed flask, 40 mL of 45% ethanol was added, and extraction performed for different times (30–150 min) at 85 °C. Secondly, we studied the influence of the concentrations of extract solvent on TFC yield. 40 mL of different concentrations (30–90%) of ethanol were added and the extraction performed for 30 min at 85 °C. Thirdly, we studied the impact of the liquid-to-solid ratio on TFC yield in the range from 10 to 50. Different volumes of 45% ethanol (10–50 mL) were added and the extraction performed for 30 min at 85 °C. Lastly, the effect of extraction temperature was investigated in the range from 75 °C to 95 °C. 40 mL of 45% ethanol was added and the extraction performed for 30 min at different temperatures (75–95 °C).

The RSM solvent extraction procedure was set as follows: *Gynura medica* leaf powder was put into a 100 mL round bottomed flask, then different concentrations of ethanol-water were added (30–60%) with different liquid to solid ratio (30–50), then put in thermostatic water bath at selected temperatures (85–95 °C) for a constant extraction period of 30 min.

All of the extracts were centrifuged at 4000 rpm for 10 min. The supernatant was collected and transferred into a 50 mL volumetric flask for the determination of TFC and anti-radical potential. 1 g of the sample was precisely weighed and used for each experiment.

### 3.5. Determination of TFC

The total flavonoid content (TFC) in the extracts was determined by using the colorimetric assay [36] with slightly modifications. Briefly, 0.3 mL solution of extracts in 45% ethanol was separately mixed with 8 mL of 10% aluminum chloride and 4 mL of 0.2 M sodium acetate. The mixed solution was then immediately diluted to volume (25 mL) with deionized distilled water and mixed thoroughly and left at room temperature for 30 min. The absorbance of the reaction mixture was measured at 350 nm by using a UV-2450 spectrophotometer (Shimadzu, Japan). Total flavonoid contents were calculated by using a kaempferol calibration curve. The calibration equation for kaempferol was *Y* = 0.04177*X* + 0.014181 (*R*^2^ = 0.9993). The results were expressed as mg kaempferol/g DM.

### 3.6. DPPH Free Radical Scavenging Activity

The antiradical activity of the extracts was evaluated by DPPH method as described by [37] with slight modification. Briefly, 0.1, 0.2, 0.3, 0.4 and 0.5 mL aliquots of 10-fold diluted extracts (concentration 2.0 mg/mL DM) were adjusted to 0.5 mL with ethanol and then mixed with 0.5 mL of 0.6 mM DPPH ethanol solution. The volume of the mixture solution was adjusted with ethanol to a final volume of 5 mL. The DPPH buffered solution used as a control was prepared by mixing the same amounts of 0.6 mM DPPH solution and 4.5 mL ethanol. After incubation in a dark place for 30 min at room temperature, the absorbance of the mixture was measured at 515 nm against ethanol as a blank using a UV-2450 spectrophotometer (Shimadzu, Japan). Each sample was measured in triplicate and averaged. The DPPH radical scavenging activity was calculated according to the following equation:

(4) $$\text{DPPH RSA}(\%)=[(A_{C}-A_{S})/A_{C}]\times 100$$

where *A*_C_ is the absorbance value of the control and *A*_S_ is the absorbance value of the added test sample solution. Trolox and quercetin were used to plot the calibration curve and the results were expressed as μmol standard reference equivalents per g dry material antioxidant capacity.

### 3.7. ABTS^+^ Cation Radical Scavenging Assay

The ABTS^+^ cation radical scavenging assay was conducted using the method described by [37]. ABTS was dissolved in water to a concentration of 7 mmol/L. ABTS^+^ was produced by reacting the ABTS stock solution with 2.45 mmol/L potassium persulfate (final concentration) and allowing the mixture to stand in the dark at room temperature for 12–16 h before use. For the test of samples, the ABTS^+^ stock solution was diluted with 80% methanol to an absorbance of 0.70 ± 0.02 at 734 nm. Aliquots of 50, 75, 100, 125 and 150 μL of 10-fold diluted extracts were adjusted to 150 μL with methanol and then mixed with 4.85 mL of diluted ABTS^+^. The ABTS^+^ solution used as a control was prepared by mixing the same amounts of ABTS^+^ solution and 150 μL methanol. The absorbance reading was taken 6 min after the initial mixing. Each sample was measured in triplicate and averaged. The ABTS^+^ cation radical scavenging activity was calculated by the same equation as for DPPH. Trolox and quercetin were used to plot the calibration curve and the results were expressed as μmol standard reference equivalents per g dry material antioxidant capacity.

### 3.8. HPLC-DAD-MS Analyses

An HPLC-DAD-ESI-MS system consisting of a Waters 2995 Series LC and ZQ-4000 Mass spectrometer (Waters, USA), equipped with a vacuum degasser, a quaternary pump, an autosampler, a thermostatted column compartment, a diode array detector (DAD) and an ion-trap mass spectrometer with electrospray ionization interface, controlled by Waters 2995 Series LC/ZQ-4000 Trap Software. Shimadzu shimpack VP-ODS (150 mm × 4.6 mm i.d., 5 μm particle size) was used for separation. Solvents for the mobile phase were water-0.1% acetic acid (A) and acetonitrile (B). The gradient elution was: 0–30 min, linear gradient 10–30% B; 30–45 min, linear gradient 30–60% B. The flow rate was 0.8 mL/min and the column was operated at 30 °C. Peaks were detected with the DAD at 347 nm and the injection volume was 20 μL. The ESI negative and positive TIC modes were used for MS detection. The *m/z* values of the monitored ions were from 100 to 800. The other parameters were as follows: capillary voltage, 3.5 kV; cone voltage, 30 V; extraction voltage, 5 V; RF voltage, 0.5 V; source temperature, 90 °C; nitrogen gas flow for desolvation, 300 L/h; and temperature of the nitrogen gas for desolvation, 350 °C.

### 3.9. HPLC-DAD Quantification

HPLC analyses were carried out on a HITACHI instrument equipped with an L-2130 binary pump, an L-2455 Diode Array Detector, an L-2300 column oven and an L-2200 automated sample Injector. Quantification was carried out using external standard method and kaempferol-3-*O*-*β*-d-gucoside was used as standard. The HPLC conditions were the same as aforementioned in Section 3.7. Analyses were carried out in triplicate and the results were expressed as mg/g DM.

## 4. Conclusions

The response surface methodology (RSM) was successfully employed to optimize the extraction of flavonoid compounds from *G. medica* leaf. The temperature and liquid-to-solid ratio were the most important factors influencing the extraction, while the time was limited to 30 min—longer extraction times significantly decreased the content of flavonoid compounds. The best combination of response function was 55% ethanol, 92 °C, and 50 (v/w) liquid-to-solid ratio with 30 min extraction time. The extracts produced using those conditions showed moderate antioxidant potential. Quercetin and kaempferol glucosides were the major flavonoid compounds present in the plant and kaempferol-3-*O*-glucoside was the predominant constituent with a quantification of 0.428 mg/g DM.

## Figures and Tables

**Table 1.** Coded and actual levels of three variables.

**Variables**	**Coded Levels of Variables**		
	−1	0	1
Ethanol concentration (*X*_1_)	30	45	60
Temperature (*X*_2_)	85	90	95
liquid-to-solid ratio (*X*_3_)	30	40	50

**Table 2.** Experimental designs using Box-Behnken and results.

**Test Run No.**	**Coded Levels of Variables**			**TFC mg kaempferol/g DM**
	*X*_1_	*X*_2_	*X*_3_	
1	1	−1	0	32.46
2	−1	1	0	32.94
3	0	−1	1	34.75
4	0	1	1	36.22
5	0	−1	−1	33.36
6	1	1	0	34.68
7	1	0	−1	32.18
8	−1	0	1	34.39
9	−1	0	−1	31.66
10	1	−1	0	32.32
11	0	1	−1	34.24
12	1	0	1	35.29
13	0	0	0	35.86
14	0	0	0	35.91
15	0	0	0	36.13

**Table 3.** Analysis of variance (ANOVA) for the regression equation.

**SD**	**DF**	**SS**	***F* value**	**Prob > *F***	**S**
Model	9	33.71	22.48	0.0016	**
*X*_1_	1	1.13	6.79	0.0480	*
*X*_2_	1	3.36	20.16	0.0065	**
*X*_3_	1	10.58	63.47	0.0005	**
*X*_1_*X*_2_	1	0.88	5.27	0.0702	
*X*_1_*X*_3_	1	0.037	0.22	0.6587	
*X*_2_*X*_3_	1	0.086	0.52	0.5044	
*X*_1_^2^	1	15.74	94.48	0.0002	**
*X*_2_^2^	1	2.37	14.22	0.0130	*
*X*_3_^2^	1	1.00	6.00	0.0580	
Lack of fit	3	0.79	12.42	0.0754	

SD: sources of deviation; DF: degree of freedom; SS: sum of squares; S: significant;

* *p* < 0.05,

** *p* < 0.01.

**Table 4.** DPPH free radical scavenging capacity.

**Final Concentration (μg/mL DM)**	**DPPH**		
	**RSA%**	**μmol Q/g DM**	**μmol T/g DM**
40	9.77 ± 1.29	50.92 ± 5.35	137.30 ± 8.98
80	15.93 ± 0.80	38.20 ± 1.65	90.03 ± 2.78
120	32.50 ± 1.20	47.83 ± 1.64	97.38 ± 2.74
160	47.41 ± 0.67	51.65 ± 0.69	99.65 ± 1.16
200	63.69 ± 1.66	54.78 ± 1.37	102.31 ± 2.30

DM: dry material; RSA: radical scavenging activity; Q: quercetin; T: trolox.

**Table 5.** ABTS^+^ cation radical scavenging capacity.

**Final Concentration (μg/mL DM)**	**ABTS^+^**		
	**RSA%**	**μmol Q/g DM**	**μmol T/g DM**
20	27.35 ± 14.0	74.98 ± 4.79	192.81 ± 10.25
30	39.70 ± 1.23	87.91 ± 3.78	209.68 ± 8.09
40	52.95 ± 0.65	94.76 ± 1.49	220.74 ± 3.19
50	66.41 ± 0.86	101.98 ± 1.59	231.15 ± 3.40
60	81.53 ± 1.31	108.21 ± 2.01	242.31 ± 4.30

DM: dry material; RSA: radical scavenging activity; Q: quercetin; T: trolox.

**Table 6.** HPLC-DAD and ESI-MS analysis of the flavonoid compounds from *G. medica* leaf extract.

**Peak No.**	**RT (min)**	**λ_max_ (nm)**	**Product Ions (ESI^−^, *m/z*)**	**Product Ions (ESI^+^, *m/z*)**	**Identification Compounds**	**Content mg/g DM**
1	12.85	264, 347	609	611, 449, 287	kaempferol-3,7-di-*O*-*β*-d-glucoside	0.155
2	19.81	254, 356	609	611, 465, 303	quercetin-3-*O*-rutinoside	0.053
3	20.96	256, 354	463	465, 303	quercetin-3-*O*-*β*-d-glucoside	0.038
4	21.61	265, 346	593	595, 449, 287	kaempferol-3-*O*-robinobioside	0.040
5	22.86	265, 347	593	595, 449, 287	kaempferol-3-*O*-rutinoside	0.102
6	24.59	265, 347	447	449, 287	kaempferol-3-*O*-*β*-d-glucoside	0.423
7	39.46	266, 367	285	287	kaempferol	0.018
